# Effect of sarcopenia on postoperative ICU admission and length of stay after hepatic resection for Klatskin tumor

**DOI:** 10.3389/fonc.2023.1136376

**Published:** 2023-03-09

**Authors:** Hyun Eom Jung, Dai Hoon Han, Bon-Nyeo Koo, Jeongmin Kim

**Affiliations:** ^1^ Department of Anesthesiology and Pain Medicine, Anesthesia and Pain Research Institute, Yonsei University College of Medicine, Seoul, Republic of Korea; ^2^ Department of Surgery, Yonsei University College of Medicine, Seoul, Republic of Korea

**Keywords:** hepatectomy, intensive care unit, Klatskin tumor, sarcopenia, perihilar cholangiocarcinoma

## Abstract

**Background:**

Hepatic resection of Klatskin tumors usually requires postoperative intensive care unit (ICU) admission because of its high morbidity and mortality. Identifying surgical patients who will benefit most from ICU admission is important because of scarce resources but remains difficult. Sarcopenia is characterised by the loss of skeletal muscle mass and is associated with poor surgical outcomes.

**Methods:**

We retrospectively analysed th.e relationship between preoperative sarcopenia and postoperative ICU admission and length of ICU stay (LOS-I) in patients who underwent hepatic resection for Klatskin tumors. Using preoperative computed tomography scans, the cross-sectional area of the psoas muscle at the level of the third lumbar vertebra was measured and normalised to the patient’s height. Using these values, the optimal cut-off for diagnosing sarcopenia was determined using receiver operating characteristic curve analysis for each sex.

**Results:**

Of 330 patients, 150 (45.5%) were diagnosed with sarcopenia. Patients with preoperative sarcopenia presented significantly more frequently to the ICU (77.3% *vs*. 47.9%, p < 0.001) and had longer total LOS-I (2.45 *vs* 0.89 days, p < 0.001). Moreover, patients with sarcopenia showed a significantly higher postoperative length of hospital stay, severe complication rate, and in-hospital mortality.

**Conclusions:**

Sarcopenia correlated with poor postoperative outcomes, especially with the increased requirement of postoperative ICU admission and prolonged LOS-I after hepatic resection in patients with Klatskin tumors.

## 1 Introduction

Klatskin tumors, also called perihilar cholangiocarcinomas, constitutes 50–70% of all biliary tract malignancies (1). Klatskin tumors originate from the biliary ductal epithelium and are located between the bifurcation of the cystic duct junction and the second-order intrahepatic bile duct branches (2). As the tumor progresses, the mass blocks the biliary tract, and patients typically present with cachexia, fatigue, and obstructive jaundice (3, 4). Although complete surgical resection is the only curative treatment for Klatskin tumors, less than half of the cases are resectable (5). In addition, this procedure is technically demanding to achieve a histologically negative margin and has high postoperative morbidity owing to the local anatomy (6, 7). Consequently, the risk of postoperative morbidity and mortality in Klatskin tumors remains high, despite many advances in surgical techniques and perioperative management (8).

Postoperative admission to the intensive care unit (ICU) in high-risk patients is effective for the prevention, early recognition, and management of severe complications, thereby reducing the mortality risk (9, 10). However, routine ICU admission after major surgery is not beneficial to all patients because of increased expenses and limited resources (11, 12). Therefore, it is important to determine the need for ICU admission in high-risk patients, as it can provide more efficient medical resources. However, identifying surgical patients who will benefit the most from intensive care remains difficult, and the identification method is not fully established. Prediction of ICU admission and length of stay has traditionally focused on the presence of comorbidities using scoring systems such as the Acute Physiology and Chronic Health Evaluation (APACHE) II, Simplified Acute Physiology Score (SAPS), or Surgical Apgar scores (13, 14). To date, patients’ nutritional and functional parameters have not been routinely evaluated or considered in the decision-making process (15).

Sarcopenia refers to the loss of skeletal muscle mass and strength associated with wasting and aging (16). Cancer patients are vulnerable to sarcopenia, as malnutrition, cancer-mediated inflammation, and inactivity may lead to loss of muscle mass and strength (17, 18). Identifying preoperative sarcopenia using computed tomography (CT) analysis is widely accepted because of its practicality (19). Cross-sectional views of the trunk provide an objective method for estimating body composition, such as muscle mass and intramuscular proportions of adipose tissue in Hounsfield units (HU), and it has many clinical implications in cancer patients (20, 21). Generally, abnormalities in these indices are associated with poor postoperative outcomes. Sarcopenia is reported as an independent risk factor for poor overall survival and disease-free survival (22, 23). A negative effect of sarcopenia on short-term outcomes in hepatic resection has been reported (24).

To date, only few studies have investigated the impact of sarcopenia on predicting ICU admission and length of ICU stay (LOS-I) for patients with Klatskin tumors who underwent hepatic resection. We retrospectively analysed the relationship between preoperative sarcopenia and postoperative ICU admission and LOS-I in patients who underwent hepatic resection for Klatskin tumors. We also investigated whether other factors, including body composition and nutritional status, had an impact on postoperative ICU admission and LOS-I. We hypothesised that preoperative sarcopenia is related to poor surgical outcomes, including increased ICU requirements.

## 2 Materials and methods

The electronic medical records of patients who underwent elective hepatic resection for Klatskin tumors at Severance Hospital in Seoul, Korea, between November 2005 and June 2022 were retrospectively reviewed. This study was approved by the Internal Review Board of Severance Hospital (approval number: 4-2022-0343). The need for informed consent was waived owing to the retrospective anonymized data of the study. The study protocol conforms to the ethical guidelines of the 1975 Declaration of Helsinki.

### 2.1 Patient characteristics

We collected data including patients’ demographics, preoperative clinical characteristics, preoperative laboratory test results, pathologic, and surgical results. Patients received the appropriate preoperative treatment, such as endoscopic biliary stenting, percutaneous transhepatic biliary drainage (PTBD), chemotherapy (CTx), and portal vein embolisation (PVE), to augment remnant liver volume. Hepatic resection included segmental resection, left or right lobectomy, and extended hepatectomy. If patients presented severe obstructive jaundice with cholangitis, we evaluated the need for preoperative biliary drainage, including PTBD, endoscopic retrograde biliary stent, and endoscopic naso-biliary drainage, depending on the specific condition. Some patients underwent neoadjuvant treatment with the purpose of downstaging in the case of borderline resectable (suspicious tumor invasion to the contralateral portal vein or hepatic artery, and a tumor located over the U-point or P-point) or locally advanced (suspicious local lymph node metastasis) Klatskin tumor (25). PVE was performed to augment the remnant liver volume, and the decision to resect the Klatskin tumor was made using a multidisciplinary approach considering vessel invasion, possibility of achieving negative margins, and future remnant liver volume (26). We collected data on surgical and oncological outcomes, including postoperative morbidity, mortality, ICU admission, length of hospital stay (LOS-H) and LOS-I, overall survival, and recurrence. Criteria for postoperative ICU admission are for patients who need intensive monitoring, treatment, and ventilator care due to medical or surgical complications such as shock, heart failure, respiratory failure, renal injury, and massive bleeding. Postoperative ICU admission was arranged during the total perioperative period with flexibility including the intraoperative situation and patients’ status in the recovery room. Patients who were not directly admitted to the ICU from the operation room but underwent delayed admission or re-admission within 7 days from the surgery date because of an emergent reason, such as complications or re-operation requiring ICU care, were also included. Therefore, the LOS-I was the sum of the total length of ICU stay if admitted within 7 days after surgery. The grade of postoperative morbidity was assigned according to the Clavien–Dindo classification (CDC) within 90 days of surgery or until the discharge date, and severe complications were defined as complications with a CDC ≥ 3 (27). Postoperative mortality was recorded during the same period. To evaluate the preoperative nutritional status of cancer patients, the prognostic nutritional index (PNI), suggested as a clinical predictor of prognosis, was calculated using collected laboratory data (28). PNI was defined as 10 × serum albumin value (g/dL) + 0.005 × lymphocyte count (/mm³), with a cut-off value for low PNI of less than 1.39 indicating nutritional impairment. Cancer recurrence and survival were determined from the time of surgery to the time of event or the most recent follow-up date.

### 2.2 Radiologic body composition evaluation

Preoperative sarcopenia was evaluated according to the obtained preoperative abdominal and pelvic CT images within 90 days before surgery which were routinely obtained to diagnose and plan treatment. The cross-sectional surface (cm²) of both psoas muscle areas (PMA) was automatically quantified at the third lumbar (L3) vertebra level using the Aquarius iNtuition Viewer (ver. 4.4.13, TeraRecon Inc., San Mateo, CA, USA) imaging server platform (29). Using this software, each surface can be automatically quantified using a particular CT attenuation range. Non-contrast-enhanced CT images were used to measure the radiation attenuation density (HU) of the muscle and adipose tissue. Using these values, muscle steatosis was evaluated using intramuscular adipose tissue content (IMAC) by dividing the CT attenuation value of the multifidus muscle (HU) by that of the subcutaneous fat HU (30). A higher IMAC indicates that skeletal muscles contain a greater amount of adipose tissue and may result in poor prognosis in several cancers (31–33). Cut-off values for high IMAC (-0.358 for men and -0.229 for women) were defined in a previous study including healthy donors for liver transplantation (34).

The measured PMA was then normalised by patient height squared (cm²/m²), which was termed the psoas muscle mass index (PMI). Optimal cut-off points for PMI data in the maximal predictive value for postoperative ICU admission were determined using receiver operating characteristic (ROC) curve analysis. Sarcopenia was determined when the patients’ PMI was lower than the sex-specific cut-off. The study population was categorised into two groups: sarcopenia and non-sarcopenia patients, and postoperative short- or long-term outcomes were compared.

### 2.3 Statistical analysis

Categorical variables are expressed as frequencies and percentages. Continuous variables are summarised as means with standard deviations (SDs). Categorical variables were analysed using the χ2 or Fisher’s exact test, and continuous variables were compared using the independent t-test or Mann–Whitney U test. Spearman’s correlation tests were used to assess the relationships between risk factors and continuous outcomes. Multivariate analysis was performed to investigate statistically significant risk factors affecting ICU admission and LOS-I using variables with *p*-values < 0.05. Survival estimates were obtained using the Kaplan–Meier survival method, and differences in overall survival (OS) and recurrence-free survival (RFS) between the groups were determined using the log-rank test. All results with *p*-values < 0.05 were statistically significant. All statistical analyses were performed using IBM SPSS Statistics for Windows, version 26.0 (IBM Corp., Armonk, NY, USA).

## 3 Results

### 3.1 Study population

A total of 330 patients with pathologically confirmed Klatskin tumors who underwent curative hepatic resection were identified during the study period. Among them, 13 patients were excluded because they underwent hepatic wedge resection (n = 2) or cooperation with other organs (n = 11). Ultimately, 317 patients were included in the analysis. The T stage was unmeasured in three patients owing to post-CTx changes in pathological specimens. The mean age of the study population was 65.6 ± 9.0 years, and 202 men (63.7%) and 115 women (36.3%) were included. The PMI (men: 6.91 *vs*. women: 3.78, *p* < 0.001) and IMAC (men: -0.44 *vs*. women: -0.32, *p* < 0.001) showed significant differences between sexes. Among them, 196 patients (61.8%) were provided ICU care within seven postoperative days, and most of them (n = 193/196, 98.4%) were directly transported from the operating theatre to the ICU. During the same period, 11 patients (3.5%) were readmitted to the ICU after being discharged from the ICU to the general ward. Among them, 4 (36.4%) developed septic shock, 2 (18.2%) underwent hepatic artery embolization or operation due to bleeding, 2 (18.2%) underwent thrombus aspiration and stent insertion due to portal vein thrombosis, 1 (9.1%) had hepatic encephalopathy, 1 (9.1%) had ventilator care due to pulmonary edema, and 1 (9.1%) experienced liver failure. Severe complications (CDC ≥ 3) were observed in 125 (39.4%) patients within 90 days of surgery, with 82 patients requiring surgical, endoscopic, or radiological intervention due to pleural effusion, pericardial effusion, bile leakage, abdominal abscess, ascites or bleeding (CDC = 3). 21 required intensive care or ventilator due to organ dysfunction like renal failure, hepatic failure, cardiogenic shock, respiratory distress, sepsis or stroke (CDC = 4). 22 patients (6.9%) died owing to postoperative mortality (CDC = 5). **Table 1** summarises patient characteristics and postoperative outcomes of the study population.

**Table 1:** Demographic and clinical characteristics of patients with Klatskin tumors who underwent hepatic resection.

	All patients (n = 317)	Men (n = 202)	Women (n = 115)	*p*-value
Preoperative				
Age (years)	65.6 ± 9.0	65.4 ± 9.0	65.9 ± 8.9	0.652
Body mass index (kg/m²)	23.42 ± 2.69	23.29 ± 2.43	23.62 ± 3.09	0.287
Psoas muscle index (cm²/m²)	5.78 ± 2.13	6.91 ± 1.72	3.78 ± 1.04	<0.001*
Sarcopenia	150 (47.3%)	106 (52.5%)	44 (38.3%)	0.015 *
Intramuscular adipose tissue content	n = 287	n = 184	n = 103	
	-0.40 ± 0.11	-0.44 ± 0.10	-0.32 ± 0.09	<0.001*
Prognostic nutritional index	44.86 ± 6.84	44.93 ± 6.81	44.73 ± 6.91	0.798
Postoperative				
Intensive care unit-admission	196 (61.8%)	127 (62.9%)	69 (60.0%)	0.613
Intensive care unit-re-admission	11 (3.5%)	7 (3.5%)	4 (3.5%)	1.000
Intensive care unit-delayed admission	3 (0.9%)	2 (1.0%)	1 (0.9%)	1.000
Length of stay-intensive care unit (days)	1.6 ± 3.4	1.6 ± 2.9	1.7 ± 4.1	0.886^†^
Postoperative length of stay- hospital (days)	24.5 ± 20.2	22.8 ± 14.2	27.4 ± 27.5	0.094^†^
Clavien–Dindo Classification ≥ 3	125 (39.4%)	78 (38.6%)	47 (40.9%)	0.693
In-hospital mortality	22 (6.9%)	16 (7.9%)	5 (4.3%)	0.492

*p < 0.05 in comparison between sexes.

^†^Compared by the Mann–Whitney U test.

ICU, intensive care unit; PMI, psoas muscle index.

### 3.2 Association between sarcopenia and postoperative short-term outcomes

The mean ± SD for PMI in men was 6.91 ± 1.72 cm²/m², whereas that in women was 3.78 ± 1.04 cm²/m² (*p* < 0.001). Sex-specific PMI cut-off values for sarcopenia were determined at 6.74 cm²/m² for men [sensitivity = 65.4%, specificity = 69.3%, area under the curve (AUC) = 0.700] and 3.39 cm²/m² for women (sensitivity = 47.8%, specificity = 76.1%, AUC = 0.609) by ROC curve analysis. Using these cut-offs, preoperative sarcopenia was observed in 106 men (52.5%) and 44 women (38.3%). The sarcopenia group had a significant lower BMI (23.0 *vs*. 23.8 kg/m², *p* = 0.004) and higher preoperative CTx incidence (12.7% *vs*. 4.8%, *p* = 0.015) than the non-sarcopenia group. Patients with sarcopenia underwent longer surgeries (550.7 *vs*. 506.7 min, *p* = 0.023) with more intraoperative bleeding (1,375 *vs*. 976 ml, *p* = 0.006), packed RBC transfusion events (44.7% *vs*. 28.1%, *p* = 0.002), and transfusion volumes (427 *vs*. 210 ml, *p* < 0.001) than those without sarcopenia.

Patients with preoperative sarcopenia presented with significantly more frequent ICU admissions within 1 week after surgery (77.3% *vs*. 47.9%, *p* < 0.001) and longer total LOS-I (2.45 *vs*. 0.89 days, *p* < 0.001) than patients without sarcopenia. Furthermore, the sarcopenia group showed significantly higher postoperative LOS-H (27.8 *vs*. 21.4 days, *p* = 0.006), severe complication rates (48% *vs*. 31.7%, *p* = 0.003), and in-hospital mortality (11.3% *vs*. 3.0%, *p* = 0.004) within postoperative 90 days than the non-sarcopenia group. Postoperative re-admission or delayed admission in ICU showed no difference between the two groups. Detailed comparisons of baseline characteristics and short-term outcomes between the sarcopenia and non-sarcopenia groups are presented in **Table 2**.

**Table 2:** Association between preoperative sarcopenia and perioperative variables.

	Sarcopenia (n = 150)	Non-sarcopenia (n = 167)	*p*-value
Preoperative			
Men	106 (70.7%)	96 (57.5%)	0.015*
Age (years)	66.5 ± 8.2	64.7 ± 9.5	0.080
Body mass index (kg/m²)	22.96 ± 2.75	23.83 ± 2.58	0.004*
American Society of Anesthesiologists score	2.4 ± 0.6	2.3 ± 0.6	0.185
Charlson comorbidity index	2.6 ± 1.3	2.5 ± 1.0	0.499
Psoas muscle index	4.76 ± 1.48	6.69 ± 2.22	<0.001*
Intramuscular adipose tissue content	-0.39 ± 0.10	-0.41 ± 0.12	0.161
Chemotherapy	19 (12.7%)	8 (4.8%)	0.015*
Portal vein embolization	43 (28.7%)	53 (31.7%)	0.553
Laboratory findings			
Haemoglobin (g/dL)	11.8 ± 1.4	12.2 ± 1.6	0.020*
Platelet count (10³/uL)	293.4 ± 101.7	301.8 ± 107.5	0.481
Prothrombin time (International normalized ratio)	1.04 ± 0.16	1.03 ± 0.11	0.421
Creatine (mg/dL)	0.80 ± 0.35	0.76 ± 0.22	0.343
Albumin (g/dL)	3.5 ± 0.4	3.7 ± 0.5	0.001*
Alanine aminotransferase (IU/L)	41.3 ± 24.6	48.6 ± 44.3	0.077
Aspartate aminotransferase (IU/L)	34.9 ± 27.9	45.1 ± 44.4	0.053
Total bilirubin (mg/dL)	1.54 ± 1.23	1.65 ± 1.98	0.552
Carbohydrate antigen 19-9 (U/mL)	918.7 ± 3118.2	566.9 ± 1743.6	0.739†
Prognostic nutritional index	44.09 ± 6.43	45.55 ± 7.14	0.058
Bismuth type			0.471
1	5 (3.3%)	5 (3.0%)	
2	14 (9.3%)	26 (15.6%)	
3a	64 (42.7%)	72 (43.1%)	
3b	24 (16.0%)	20 (12.0%)	
4	43 (28.7%)	44 (26.3%)	
Intraoperative			
Operation type			0.188
Liver lobectomy	119	118	
Extended hepatectomy	22	37	
Central lobectomy or segment resection	9	12	
Tumor stage (n = 314)	n = 148	n = 166	0.281
T1	17 (11.5%)	14 (8.4%)	
T2	97 (65.5%)	125 (75.3%)	
T3	26 (17.6%)	22 (13.3%)	
T4	8 (5.4%)	5 (3.0%)	
Lymph node metastasis	51 (34.0%)	57 (34.1%)	0.637
Resection margin negative	116 (77.3%)	118 (70.7%)	0.286
Mass size (cm)	2.8 ± 1.4	2.9 ± 1.3	0.576
Operation time (min)	550.7 ± 172.7	506.7 ± 169.7	0.023*
Estimated blood loss (cc)	1375 ± 1423	976 ± 1119	0.002*^†^
Packed RBC transfusion	67 (44.7%)	47 (28.1%)	0.002*
Transfusion amount (cc)	427 ± 838	210 ± 577	<0.001^†^
Postoperative			
Intensive care unit admission	116 (77.3%)	80 (47.9%)	<0.001*
Intensive care unit re-admission	8 (5.3%)	3 (1.8%)	0.124
Intensive care unit delay-admission	2 (1.3%)	1 (0.6%)	0.605
Length of stay-intensive care unit (days)	2.5 ± 4.5	0.9 ± 1.4	<0.001*^†^
Length of stay-hospital (days)	27.8 ± 25.4	21.4 ± 13.1	0.006*^†^
Clavien–Dindo classification ≥ 3	72 (48.0%)	53 (31.7%)	0.003*
Pleural effusion requiring intervention	25 (16.7%)	23 (13.8%)	
Reoperation	9 (6.0%)	7 (4.2%)	
Bile leakage or ascites requiring intervention	8 (5.3%)	6 (3.6%)	
Sepsis	3 (2.0%)	4 (2.4%)	
In-hospital mortality	17 (11.3%)	5 (3.0%)	0.004*

*p < 0.05 in comparison with the non-sarcopenia group.

^†^Compared by the Mann–Whitney U test.

Abbreviations: BMI, body mass index; ICU, intensive care unit; RBC, red blood cell.

Multivariate analysis showed that sarcopenia was significantly associated with postoperative ICU admission [adjusted odds ratio (OR): 2.461, *p* = 0.006] and prolonged LOS-I (B: 0.957, *p* = 0.008). Other factors associated with postoperative ICU admission in the multivariate analysis were serum carbohydrate antigen (CA) 19-9 levels, operation time, and intraoperative packed RBC transfusion events. Alternatively, BMI, Charlson comorbidity index, T stage 3 or 4, operation time, and intraoperative packed RBC transfusion events were significantly associated with LOS-I in multivariate analysis (**Tables 3**, **4**).

**Table 3:** Multivariate logistic regression analysis for risk factors of postoperative ICU admission.

Variable	OR	95% CI		*p*-value
		*Lower limit*	*Upper limit*	
Men	1.109	0.554	2.222	0.770
Age (years)	0.998	0.961	1.037	0.919
Body mass index (kg/m²)	0.964	0.841	1.106	0.604
Sarcopenia	2.461	1.289	4.697	0.006*
Intramuscular adipose tissue content > -0.358 (men), > -0.229 (women)	2.259	0.743	6.873	0.151
Prognostic nutritional index < 40	0.865	0.220	3.394	0.835
American Society of Anesthesiologists score	1.731	0.815	3.675	0.153
Charlson comorbidity index	0.885	0.583	1.342	0.565
Pre-operative chemotherapy	2.214	0.582	8.428	0.244
Haemoglobin < 13.0 (men, g/dL) < 11.4 (women)	1.377	0.703	2.694	0.351
Prothrombin time (international normalized ratio) > 1.12	2.320	0.944	5.700	0.066
Preoperative albumin < 3.3 (g/dL)	2.014	0.481	8.438	0.338
Carbohydrate antigen (CA) 19-9 (U/mL)	1.000	1.000	1.001	0.028*
T stage 3 or 4	1.599	0.637	4.009	0.317
Bismuth type 2	0.682	0.286	1.625	0.388
Packed RBC transfusion	4.027	1.813	8.945	0.001*
Operation time (min)	1.006	1.003	1.008	0.000*

*p< 0.05 in comparison with the non-sarcopenia group. CI, confidence interval; ICU, intensive care unit; OR, odds radio; RBC, red blood cell.

**Table 4:** Multivariate linear regression analysis for risk factors of postoperative length of ICU stay (days).

Variable	B	95% CI (B)		ß	*p*-value
		*Lower limit*	*Upper limit*		
Men	-0.353	-1.062	0.356	-0.050	0.328
Age (years)	0.002	-0.038	0.041	0.004	0.939
Body mass index (kg/m²)	-0.143	-0.277	-0.009	-0.114	0.036*
Sarcopenia	0.957	0.252	1.663	0.142	0.008*
Charlson comorbidity index	0.409	0.084	0.734	0.139	0.014*
Pre-operative chemotherapy	0.946	-0.319	2.210	0.078	0.142
Platelet count < 150 (10³/uL)	1.643	-0.137	3.423	0.093	0.070
Prothrombin time (international normalized ratio) > 1.12	0.662	-0.259	1.583	0.072	0.158
Total bilirubin (mg/dL)	0.194	-0.012	0.400	0.096	0.065
T stage 3 or 4	1.507	0.624	2.391	0.176	0.001*
Packed RBC transfusion	0.953	0.132	1.774	0.136	0.023*
Operation time (min)	0.004	0.001	0.006	0.186	0.001*

*p < 0.05 in compared with the non-sarcopenia group.

CI, confidence interval; ICU, intensive care unit; RBC, red blood cell.

### 3.3 Impact of the nutritional status and body composition on short-term outcomes

Non-contrast-enhanced CT images were available for 285 patients, and a high IMAC was observed in 12.0% (n = 38/285) of the patients. A strong linear correlation was noted between PMI and IMAC (correlation coefficient; -0.414, *p* < 0.001), and a high IMAC was more frequent in sarcopenia patients (17.4% *vs*. 9.4%, *p* = 0.046) than in patients without sarcopenia. Preoperative nutritional assessment *via* laboratory data revealed that patients with sarcopenia had lower serum haemoglobin and albumin levels than patients without sarcopenia. A low PNI was noted in 74 (23.3%) patients and was significantly more frequent in the sarcopenia group (28.7% *vs*. 18.6%, *p* = 0.034) than in the non-sarcopenia group. A high IMAC (81.6% *vs* 58.6%, *p* = 0.007) and low PNI (73.0% *vs* 58.4%, *p* = 0. 024) were significantly associated with an increased postoperative ICU admission rate.

### 3.4 Survival analysis

**Figure 1:**
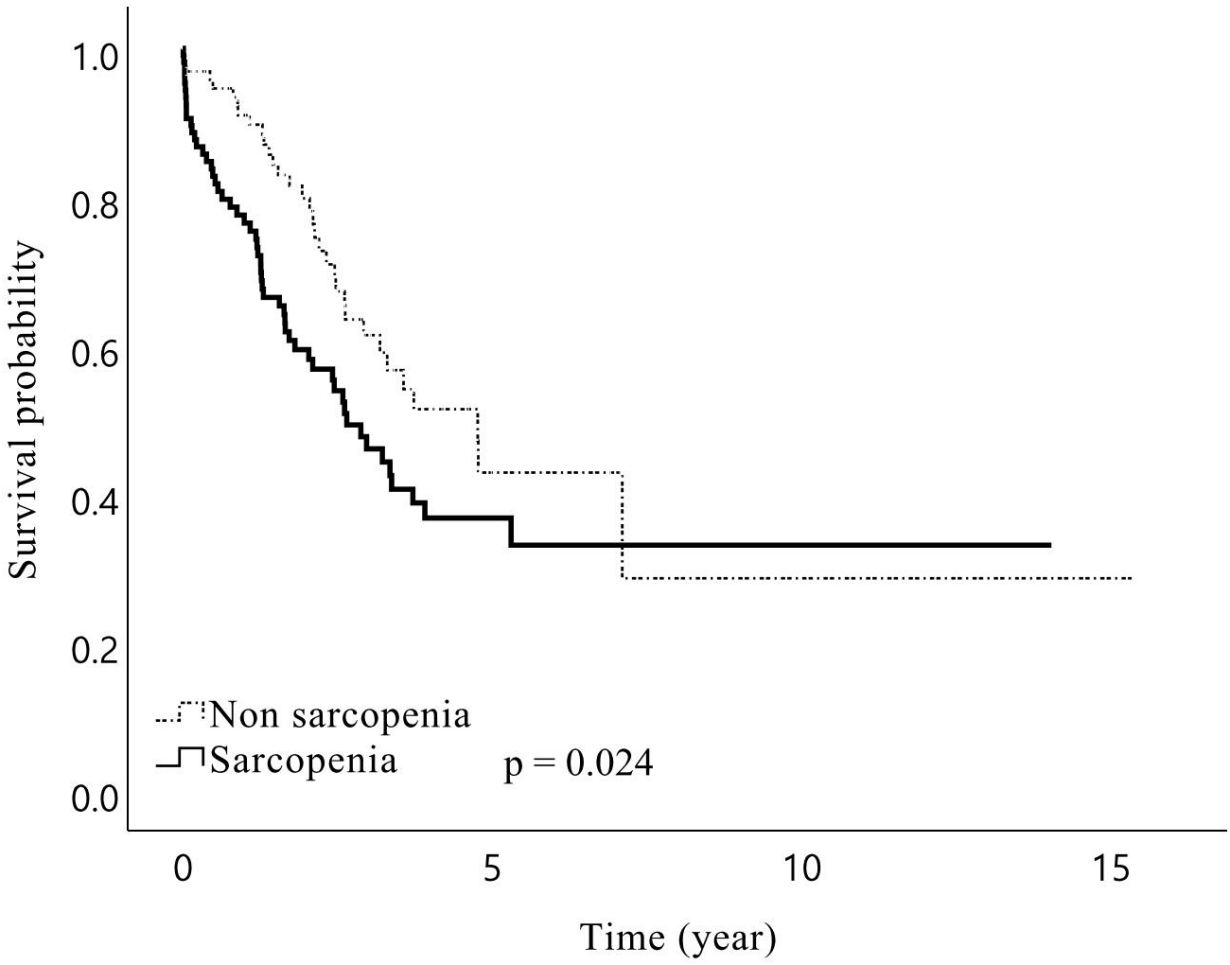
Kaplan–Meier curves for overall survival after hepatic resection of Klatskin tumors for male patients stratified by presence of preoperative sarcopenia. Overall survival for male patients with sarcopenia following surgery of Klatskin tumors was shorter than for those without sarcopenia.

**Figure 2:**
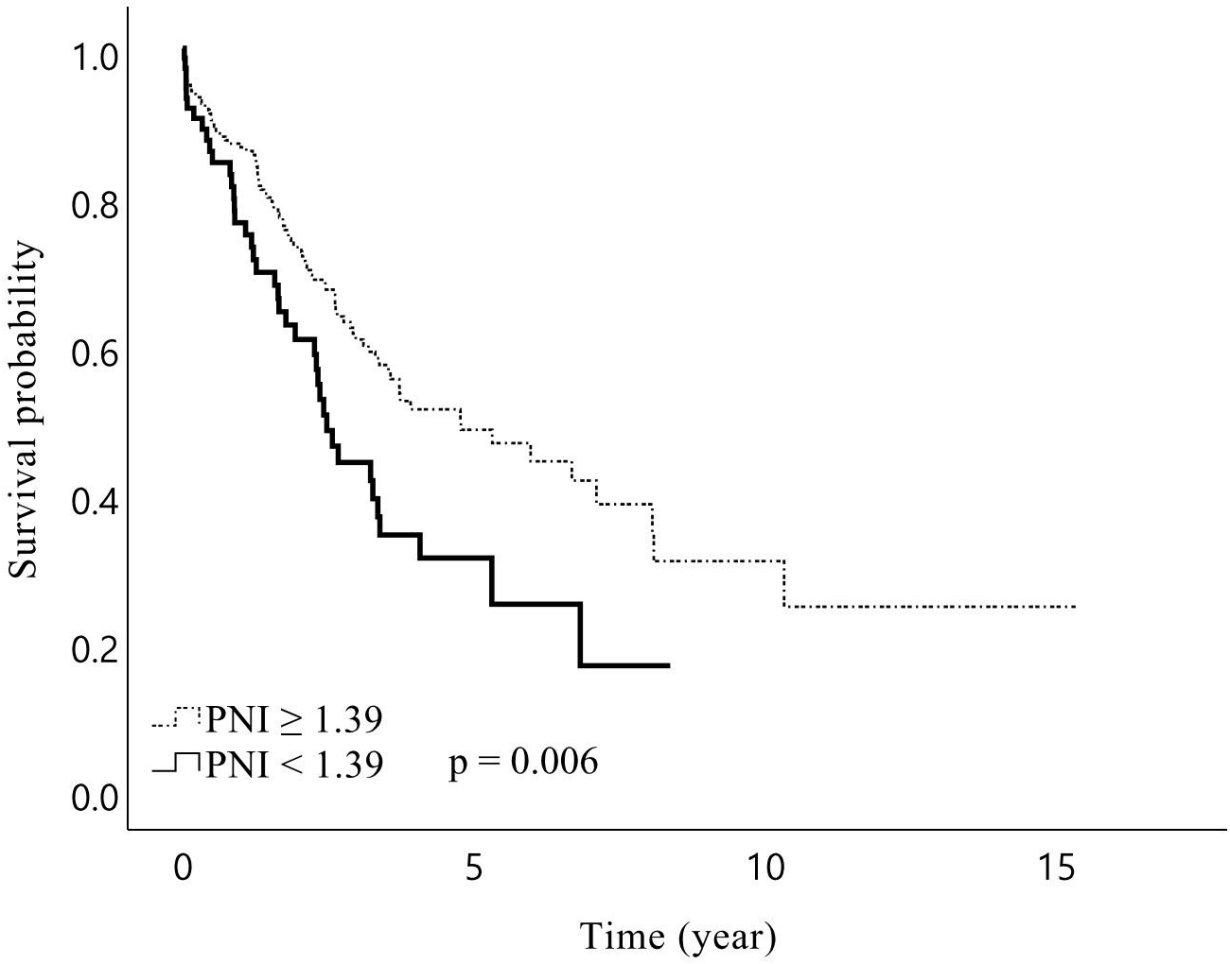
Kaplan–Meier curves for overall survival after hepatic resection of Klatskin tumors stratified by prognostic nutritional index (PNI) Preoperative PNI lower than 1.39 was associated with a shorter overall survival for patients with Klatskin tumors following surgery.

In total, the median follow-up duration among the living patients was 44.3 months, and 181 (57.1%) patients experienced recurrence during the study period. The 1-, 3-, and 5-year OS rates of the patients were 84%, 57%, and 44%, respectively, while RFS rates were 74%, 39%, and 25%, respectively. The median RFS was 27.8 months in patients with sarcopenia and 20.3 months in patients without sarcopenia, but the difference was not statistically significant (*p* = 0.609). However, sarcopenia patients showed a trend towards a lower OS than non-sarcopenia patients, with borderline significance (median 38.7 *vs* 57.3 months, *p* = 0.075). In the subgroup analysis, male patients with sarcopenia had significantly lower OS than those without sarcopenia (median 31.8 *vs* 57.2 months, *p* = 0.024, **Figure 1**). Further analysis of patients’ IMAC and PNI did not yield significant impacts on RFS and OS, except that patients with a low PNI were associated with lower OS (median 29.7 *vs* 57.2 months, *p* = 0.006, **Figure 2**) than patients with a normal PNI.

## 4 Discussion

We aimed to investigate the clinical impact of sarcopenia on the surgical outcomes of patients who underwent hepatectomy for Klatskin tumors. The results showed that preoperative sarcopenia increased the ICU admission rate and prolonged ICU stay after hepatic resection in patients with Klatskin tumors. Additionally, sarcopenia patients showed significantly higher postoperative severe morbidity and mortality than non-sarcopenia patients. Our study implies that sarcopenia patients present a higher necessity of being admitted to the ICU postoperatively and should be given priority among high-risk patients who underwent major surgeries.

Sarcopenia, a physiological syndrome characterised by a combination of low muscle mass and low muscle function, has been proposed as a factor that increases perioperative risk for adverse clinical outcomes (35). In particular, frequent obstructive jaundice in patients with Klatskin tumors makes them vulnerable to poor oral intake and decreased activity, which consequently induce sarcopenia (36, 37). Among many previous definitions, the cut-offs for diagnosing sarcopenia for Klatskin tumor differed depending on the studies (range of PMI cut-off for men: 4.77 – 8.60 cm²/m² and for women: 3.38 – 6.04 cm²/m²) (38). We used the normalised PMA which is widely used to diagnose sarcopenia, and the cut-off value was determined by ROC curve analysis by taking the rate of ICU admission as an indicator for predictive validity to determine the optimal cut-off value for each sex (6.74 cm²/m² for men and 3.39 cm²/m² for women). Sarcopenia was present in 47.4% of our population, which is consistent with previous reports stating that sarcopenia was common among patients with Klatskin tumors, occurring in more than 30% of patients (29).

In addition, we analysed other parameters, such as body composition and nutritional status. Through image analysis, we observed that muscle steatosis determined by a high IMAC was associated with postoperative ICU admission following hepatic resection of Klatskin tumors. This also corresponds with our earlier observations which showed that a poor preoperative nutritional status of patients based on the calculated low PNI increased ICU requirements. Furthermore, a high IMAC and low PNI were more frequent among patients with sarcopenia than among those without sarcopenia, which indicates their poor nutritional status and low muscle density. This result suggests that preoperative sarcopenia is strongly related to muscle steatosis and poor nutritional status with weight loss, indicating increasing requirements for postoperative ICU care.

Our findings also support those of previous studies, as we showed that sarcopenia was associated with increased operation time, intraoperative blood loss, packed RBC transfusion, and poor overall short-term prognosis (29, 39). Moreover, sarcopenia showed poor OS which was consistent with findings of previous studies, while we did not observe any relevance to RFS (38). It can be interpreted that our cut-off was based on the ROC curve analysis of ICU admission. However, intraoperative transfusion and longer operation time were significantly related to both postoperative ICU admission and LOS-I in multivariate analysis. Poor nutritional status and intraoperative packed RBC transfusion are also associated with poor OS in cholangiocarcinoma (40, 41). In addition, prolonged operation time is a known risk factor for postoperative complications, which can help to interpret our results (42).

To the best of our knowledge, the current study is the first to analyse the impact of preoperative sarcopenia on postoperative ICU care frequency and duration. Postoperative ICU care is beneficial for the management and early detection of severe complications. However, unnecessary postoperative ICU admission only for surveillance is not appropriate considering low cost-effectiveness and increased ICU-related complications, such as infection or delirium (43, 44). These issues have been considered during the COVID-19 pandemic which induced a shortage of critical care beds and staff (45). Likewise, unpredicted, prolonged ICU stays can negatively affect ICU bed resources and may alter other operational schedules (46). Therefore, filling surgical requests for ICU preparation should consider objective triage to maximise patient outcomes and medical resources (47). Although some scoring systems have been developed to aid the preoperative determination of surgical candidates for ICU care, they are insufficient to provide adequate information regarding the risk for an individual patient (13, 48). We suggest that physicians diagnose sarcopenia using preoperative CT images and use it as a parameter in predicting postoperative ICU requirements for patients with Klatskin tumors.

The current study has some limitations. 1) Our study has the possibility of temporal or selection bias. Our data were collected from a single institution, and the study population was limited to East Asians (Koreans) who can undergo surgery. Furthermore, we could not measure other functional parameters of sarcopenia, such as handgrip strength, walking speed, and low physical activity, owing to the retrospective nature of the study. 2) Chronological improvements in surgical techniques and diversity of surgeons were not considered during the long-term study period. 3) Possible differences as a consequence of using different CT scanners and scanning protocols in various periods or hospitals could not be precluded. In the future, multicentre, large, prospective studies may be required to verify this result. Together, skeletal muscle mass and function should be evaluated to diagnose sarcopenia. Further randomised controlled trials should be conducted to investigate whether improving muscle mass and quality before surgery through rehabilitation or nutritional support has the potential to reduce postoperative ICU admission and length of stay (49, 50).

In conclusion, this study showed that sarcopenia was correlated with poor postoperative outcomes, especially with the increased requirement of postoperative ICU admission and prolonged LOS-I after hepatic resection for patients with Klatskin tumors. The evaluation of preoperative sarcopenia could help predict the postoperative outcomes of such patients.

## Data availability statement

The raw data supporting the conclusions of this article will be made available by the authors, without undue reservation.

## Ethics statement

The studies involving human participants were reviewed and approved by Internal Review Board of Severance Hospital. The ethics committee waived the requirement of written informed consent for participation.

## Author contributions

All authors contributed to conception and design of the study. HJ: data collection, data analysis, and writing of the first draft. DH: patient recruitment, manuscript review and critique. BK: patient recruitment, and data collection. JK: computational analysis methodology, manuscript review and critique. All authors contributed to the article and approved the submitted version.
