# Corrigendum: Effect of sarcopenia on postoperative ICU admission and length of stay after hepatic resection for Klatskin tumor

**DOI:** 10.3389/fonc.2024.1497003

**Published:** 2024-10-18

**Authors:** Hyun Eom Jung, Dai Hoon Han, Bon-Nyeo Koo, Jeongmin Kim

**Affiliations:** ^1^ Department of Anesthesiology and Pain Medicine, Anesthesia and Pain Research Institute, Yonsei University College of Medicine, Seoul, Republic of Korea; ^2^ Department of Surgery, Yonsei University College of Medicine, Seoul, Republic of Korea

**Keywords:** hepatectomy, intensive care unit, Klatskin tumor, sarcopenia, perihilar cholangiocarcinoma

In the published article, there was an error in the IRB approval statement in **Materials and methods**. The incorrect IRB approval number was provided. The correct IRB statement appears below.

“Internal Review Board of Severance Hospital (approval number: 4-2022-0343).”

The authors apologize for this error and state that this does not change the scientific conclusions of the article in any way. The original article has been updated.

